# IL-23 inhibitor treatment of immune checkpoint inhibitor-associated psoriasis: Case series and review of literature

**DOI:** 10.1016/j.jdcr.2024.09.027

**Published:** 2024-11-10

**Authors:** Natalie Bourand, Drew Kuraitis, Bethany Lema, Susan Pei

**Affiliations:** a University of Illinois College of Medicine, Chicago, Illinois; b Department of Dermatology, Roswell Park Comprehensive Cancer Center, Buffalo, New York; c Department of Dermatology, Tulane University, New Orleans, Louisiana

**Keywords:** cutaneous immune-related adverse events, guselkumab, IL-23 inhibitor, immune checkpoint inhibitor, nivolumab, PD-1 inhibitor, PD-L1 inhibitor, pembrolizumab, psoriasis, risankizumab

## Introduction

Immune checkpoint inhibitors (ICI) are monoclonal antibodies that enhance T-lymphocyte anti-tumor response through inhibiting programmed cell death protein 1 (PD-1), programmed cell death ligand 1 (PD-L1), or cytotoxic lymphocyte-associated antigen-4.^1^ Cutaneous immune-related adverse events (cirAEs) are common. Psoriasis, a common cirAE of ICIs (3.8%), presents de novo or as exacerbation, with approximately 21% requiring systemic therapy.^2^^,^^3^ There is no standardized recommendation for systemic therapy, particularly for biologics like interleukin-23 (IL-23) inhibitors. Here, we report 2 patients with PD-L1/PD-1 inhibitor-induced plaque, nail and joint psoriasis successfully treated with IL-23 inhibitors risankizumab and guselkumab.

## Case 1

**Fig 1:**
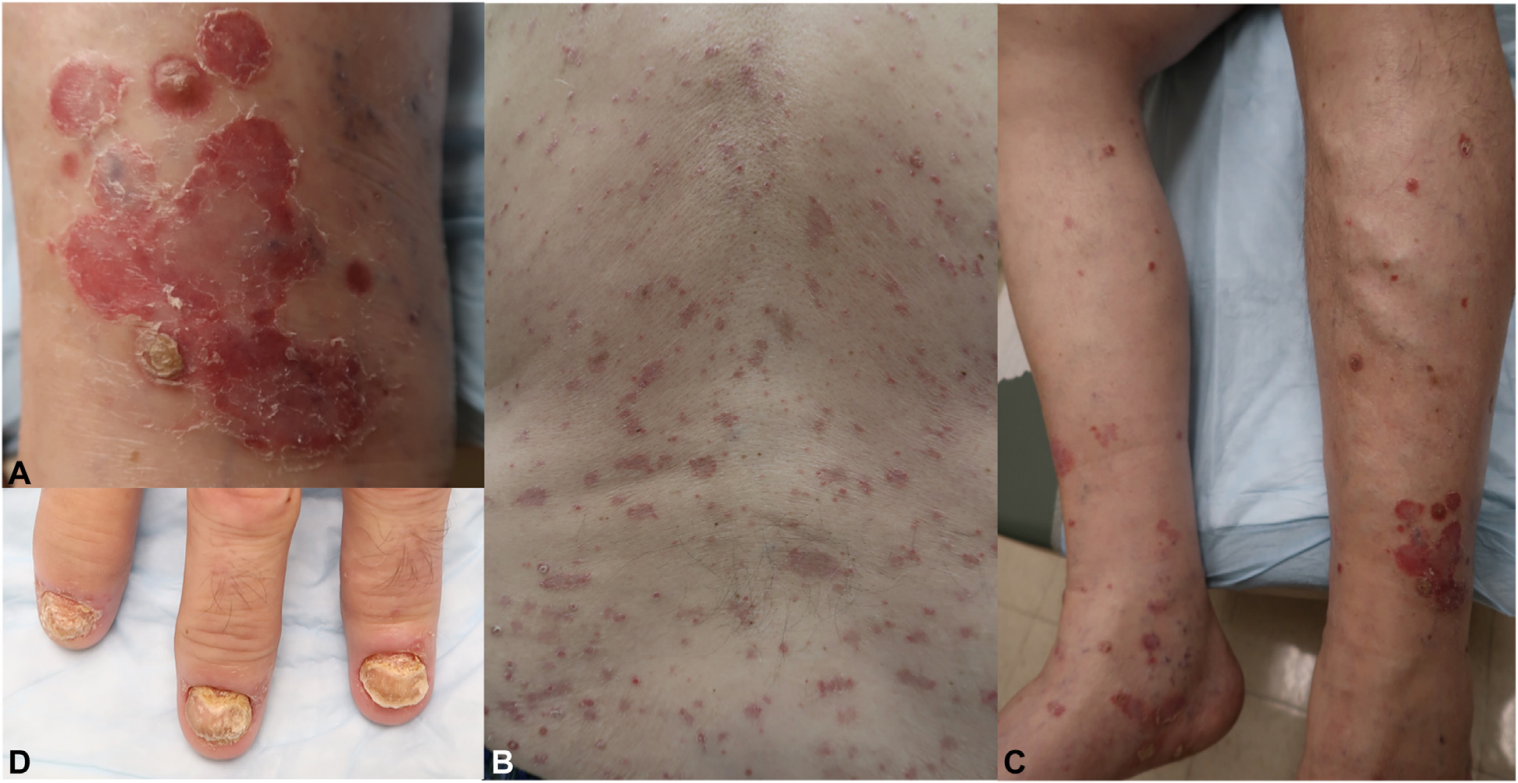
PD-1 inhibitor-induced plaque and nail psoriasis with erythematous, well demarcated scaly plaques (**A**-**C**) and onychodystrophy of fingernails (**D**).

**Fig 2:**
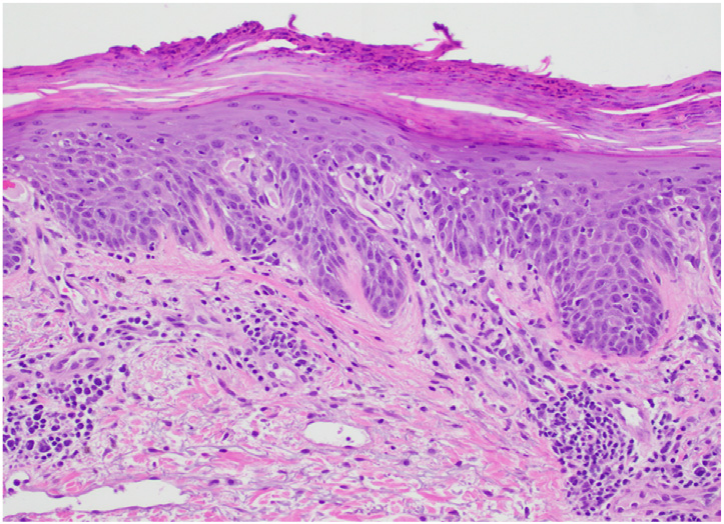
Shave biopsy demonstrating acanthosis, hypogranulosis, and parakeratosis with mounds of neutrophils in the stratum corneum. (Hematoxylin and eosin, 200×)

**Fig 3:**
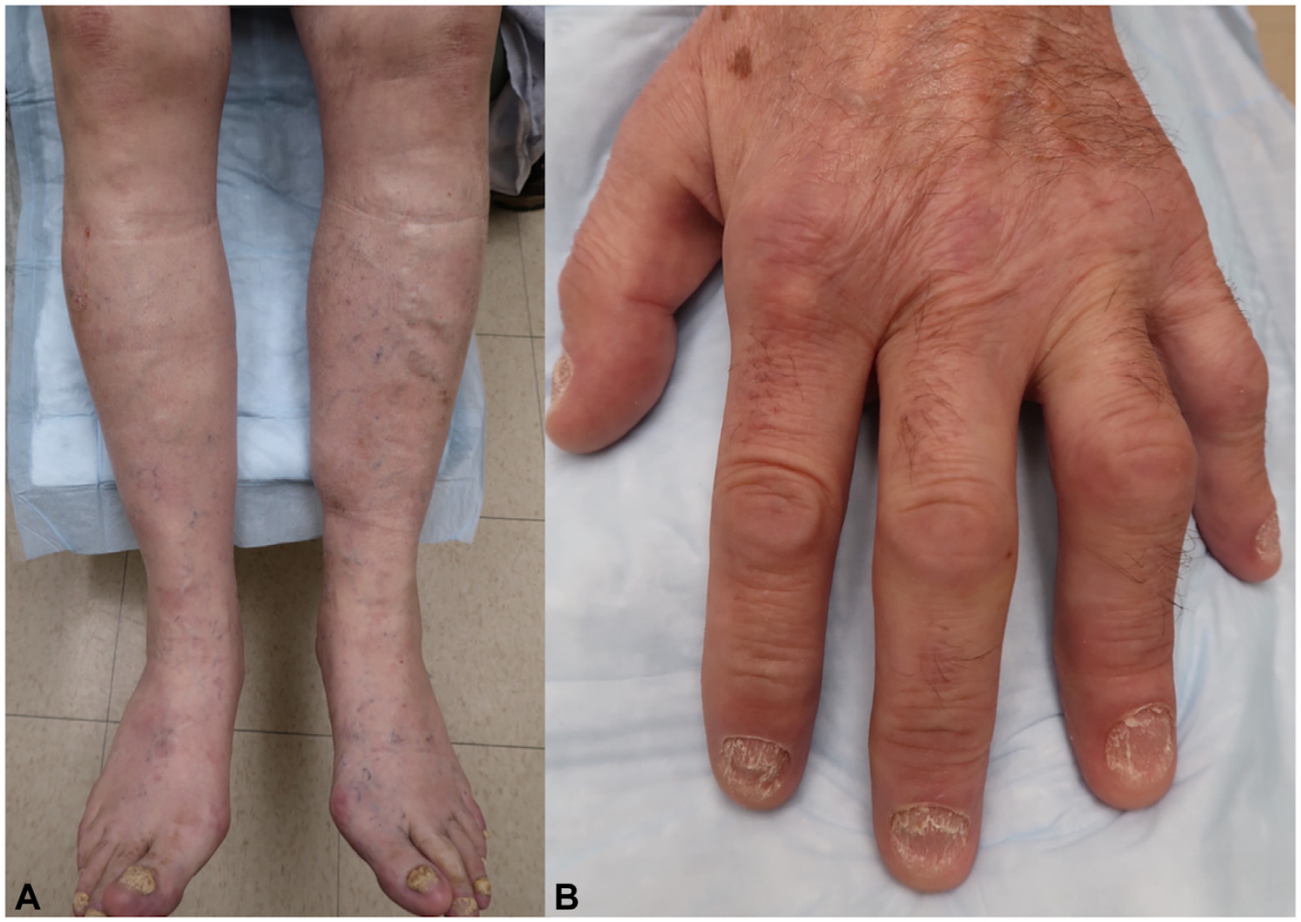
Rapid improvement of PD-1 inhibitor-induced psoriasis after 2 loading doses of risankizumab, with near complete skin clearance (**A**) and significant improvement of onychodystrophy (**B**).

A 67-year-old male with metastatic renal cell carcinoma and no psoriasis history presented with rash after 6 cycles of nivolumab. Physical exam showed erythematous, well demarcated papules and plaques with silvery scale (30% total body surface area [TBSA]) throughout the chest, back, extensor arms, umbilicus, leg, dorsal feet, and glans penis (Fig 1, *A*-*C*) with grade 2 severity (using the common terminology criteria for adverse events, common terminology for cutaneous adverse events [CTCAE]). All nails were dystrophic with yellowing and thickening (Fig 1, *D*). He reported hand and knee joint stiffness that improved with movement. Biopsy demonstrated epidermal acanthosis with hypogranulosis, mild spongiosis and parakeratosis with neutrophils in the stratum corneum (Fig 2). A diagnosis of PD1 inhibitor-induced plaque, nail and joint psoriasis was made. Nivolumab was discontinued as psoriasis progressed to grade 3 with stable tumor response. Symptoms lacked improvement with apremilast, prednisone and topical steroids. Risankizumab 150 mg standard dosing was started, rapidly improving joint symptoms with near complete clearance of all skin lesions (Fig 3, *A*) and improved onychodystrophy (Fig 3, *B*). He continued risankizumab 150 mg every 12 weeks maintenance dose, but developed tumor progression 4 months later, starting batiraxcept and cabozantinib. He continued risankizumab, sustaining psoriatic improvement for 3 years.

## Case 2

A 57-year-old male with metastatic hepatocellular carcinoma presented for rash after one cycle of pembrolizumab and sorafenib. He was previously on atezolizumab and bevacizumab, discontinued after 8 cycles due to hand and leg dermatitis. He noted the prior rash was similar to his current one, though he did not know the previous diagnosis. Physical exam showed well demarcated thick plaques with silvery scale on elbows, legs and feet (8% TBSA, grade 1) with joint pain and stiffness in legs, feet and shoulders. He deferred skin biopsy. Based on his rash onset with atezolizumab and rapid recrudescence with PD-1 inhibitor pembrolizumab, classic cutaneous morphology and psoriatic arthritis diagnosis, he was diagnosed with PD-L1/PD-1-induced psoriasis/psoriatic arthritis. Pembrolizumab and sorafenib were held; he had moderate improvement with apremilast. Tumor progression led to resumption of pembrolizumab and sorafenib, causing psoriatic symptoms to flare to grade 3, affecting daily activities. Pembrolizumab, sorafenib, and apremilast were discontinued and he started guselkumab 100 mg loading doses at 0 and 4 weeks. At 4 weeks, he had significant improvement of psoriasis (5% TBSA) with grade 1 joint symptoms. Tumor progression continued, and he has since received multiple oral chemo- and targeted therapies. His psoriatic disease remained well controlled 1 year after guselkumab initiation.

## Discussion

Topical corticosteroids are the most common therapy used for ICI-associated psoriasis.^2^ Systemic treatments include systemic steroids, acitretin, cyclosporine, apremilast, and biologics, but standardized recommendations are lacking. European Society for Medical Oncology guidelines for managing ICI induced psoriasis indicate systemic steroids should be used cautiously due to risk of rebound flare; topical steroids and vitamin D, systemic retinoids, IL-12 and IL-23 inhibitors should be considered first.^4^ Due to immunosuppression, there is concern that systemic steroids, cyclosporine, and tumor necrosis factor alpha inhibitors may interfere with ICI anti-tumor activity or exacerbate active malignancy. Apremilast, a phosphodiesterase 4 inhibitor, exerts anti-inflammatory, not immunosuppressive, effects and has been reported in limited cases to be efficacious in ICI-induced psoriasis^5^; however, our patients did not respond to apremilast.

Psoriasis pathogenesis involves IL-23/IL-17 dysregulation, where IL-23 activates Th17 cells, driving keratinocyte hyperproliferation resulting in psoriasis plaques.^6^ Epidermal keratinocytes and dermal macrophages may additionally produce IL-23, sustaining psoriatic lesions through a feed forward response. PD-1 inhibitor-associated psoriasis shares features with idiopathic psoriasis, with dendritic cells infiltrating lesions and producing IL-23, IL-17 and other cytokines.^7^ Therefore, inhibiting IL-23 and IL-17 may be reasonable treatment for PD-1/PD-L1-associated psoriasis.

IL-23 inhibitors for cutaneous psoriasis like guselkumab, risankizumab, and tildrakizumab are promising, though reports are limited (Table I).^8^, ^9^, ^10^, ^11^, ^12^ Including our 2 patients, a total of 10 patients with ICI-associated psoriasis have been treated with risankizumab (*n* = 6) and guselkumab (*n* = 4). IL-23 inhibitor therapy appears effective, with all patients achieving clearance or significant improvement of lesions, though with variable joint symptom response.

**Table I:** Reported cases of immune checkpoint inhibitor-associated psoriasis treated with IL-23 inhibitors

	Case	Sex	Age	Cancer	Cancer treatment	Cancer therapy course	Psoriasis subtype	History of psoriasis prior to ICI therapy	Prior psoriasis treatment	IL-23 inhibitor	Response to IL-23 inhibitor	Follow up after IL-23 inhibitor initiation	Cancer outcome
1	Glinos et al 2021^8^	Female	61	Stage IIIB malignant melanoma	Surgical resection, nivolumab	Nivolumab permanently discontinued despite clearance of rash	Skin psoriasis	No	Prednisone, acitretin	Risankizumab	Clearance of psoriasis	1 y	Tumor remission
2	Takeda and Yanagitani 2022^9^	Male	64	Stage IIIC lung cancer	Chemoradiotherapy, carboplatin and paclitaxel, durvalumab	Permanently discontinued	Plaque psoriasis, nail psoriasis, psoriatic arthritis	No	Prednisolone, celecoxib (for psoriatic arthritis), calcipotriol and betamethasone diproprionate ointment	Guselkumab	Clearance of plaque psoriasis and no joint symptoms, residual toenail psoriasis (CPDAI score 0)	8 mo	Not reported
3	Gargiulo et al 2023^10^	Male	28	Stage IV metastatic malignant melanoma	Ipilimumab, IL-2, pembrolizumab	Discontinued ipilimumab and IL-2; resumed pembrolizumab	Psoriasis vulgaris, palmoplantar psoriasis	Yes	Topical steroids	Risankizumab	Complete resolution of psoriasis	28 wk	Not reported
4	Fournier et al 2023^11^	Male	52	Stage IV nonsmall cell lung adenocarcinoma	Binimetinib and pembrolizumab, carboplain, pemetrexed and pembrolizumab	Discontinued binimetinib and pembrolizumab; resumed carboplatin, and pemetrexed with pembrolizumab	Plaque psoriasis, psoriatic arthritis	Yes	Calcipotriene and betamethasone diproprionate foam	Guselkumab	Good response for skin psoriasis, partial response for psoriatic arthritis	9 mo for guselkumab; after 9 mo guselkumab discontinued and switched to methotrexate for psoriatic arthritis	Partial tumor response
5	Fournier et al 2023^11^	Female	58	Metastatic melanoma	Dabrafenib and trametinib, pembrolizumab, encorafenib and binimetinib, radiation therapy	Discontinued dabrafenib and trametinib, started pembrolizumab then discontinued; gencorafeni and binimetinib started then discontinued	Plaque psoriasis, psoriatic arthritis	Yes	Calcipotriene and betamethasone diproprionate foam, tacrolimus ointment, clobetasol ointment	Guselkumab	Excellent response for skin psoriasis, no sign of psoriatic arthritis	34 mo	Deep partial response for 2 y, then progression of disease and death
6	Fournier et al 2023^11^	Male	60	Metastatic melanoma	Ipilimumab and nivolumab, nivolumab infliximab, dabrafenib and trametinib, surgery, radiation therapy	Discontinued ipilimumab and nivolumab; nivolumab restarted then permanently discontinued; infliximab discontinued; dabrafenib and trametinib continued	Guttate psoriasis, then plaque-typepsoriasis, palmoplantar pustulosis, psoriatic arthritis	Yes and positive family history (sister)	Calcipotriene and betamethasone diproprionate foam	Risankizumab	Excellent response for plaque-typepsoriasis, good response for palmoplantar pustulosis	5 mo	Brain metastases progression
7	Young et al 2023^12^	Not reported	Not reported	Recurrent ovarian cancer	Pembrolizumab	Held then resumed pembrolizumab	Skin psoriasis	Not reported	Acitretin	Risankizumab	Significant improvement	98 d	Stable tumor response
8	Young et al 2023^12^	Not reported	Not reported	Hepatocellular carcinoma	Pembrolizumab	Continued pembrolizumab with risankizumab	Eczema that then converted to psoriasis	Not reported	Prednisone, topical steroids	Risankizumab	Significant improvement	709 d	Stable tumor response
9	Current case series	Male	67	Metastatic renal cell carcinoma	Pazopanib and bevacizumab, nivolumab, batiraxcept and cabozantinib	Discontinued nivolumab; batiraxcept and cabozantinib started after risankizumab	Plaque psoriasis, nail psoriasis, psoriatic arthritis	No	Apremilast, prednisone, topical steroids	Risankizumab	Complete clearance of plaque psoriasis, improvement of nail psoriasis, intermittent residual mild joint pain	7 mo	Tumor progression 4 mo after risankizumab
10	Current case series	Male	57	Metastatic hepatocellular carcinoma	Atezolizumab and bevacizumab, pembrolizumab and sorafenib, ramucirumab, cabozantinib	Discontinued atezolizumab and bevacizumab; pembrolizumab and sorafenib held, restarted, and discontinued; ramucirumab and cabozantinib continued	Plaque psoriasis, psoriatic arthritis	No	Apremilast, topical steroids	Guselkumab	Significant improvement of plaque psoriasis and psoriatic arthritis	1 y	Tumor progression prior to and during guselkumab therapy

IL-23 inhibitor therapy safety in malignancy remains unclear. Five reported patients experienced partial tumor response and/or progression on IL-23 inhibitors, though unclear whether these biologics exert effects on the tumors (Table I). In 17 phase II and III trials of risankizumab, of 10 cancer patients, 8 remained in remission and 2 experienced recurrence.^11^ CorEvitas data on guselkumab, including 4400 patients with psoriatic disease, demonstrated favorable safety with stable cancer rates up to 5 years.^13^ However, this data include patients with cancer histories more than 5 years prior to enrollment; safety in patients with active malignancy is less documented. Animal models show mixed results, but one study found IL-23-deficient, melanoma-grafted mice exhibited resistance to melanoma-induced lung metastases.^14^ Whether ICI may be resumed after treatment of cirAEs depends on multiple factors, including prior cirAEs severity and potential ICI benefit, requiring multidisciplinary discussion.^4^

In summary, we present 2 additional cases of PD-L1/PD-1 inhibitor-induced plaque, nail, and joint psoriasis successfully treated with risankizumab and guselkumab. Although IL-23 inhibitors appear efficacious in treating PD-L1/PD-1 inhibitor-induced psoriasis, additional studies are needed, particularly regarding safety in cancer patients.

## Conflicts of interest

None disclosed.
